# Opposing Effects of Oxygen Regulation on Kallistatin Expression: Kallistatin as a Novel Mediator of Oxygen-Induced HIF-1-eNOS-NO Pathway

**DOI:** 10.1155/2017/5262958

**Published:** 2017-12-13

**Authors:** Julie Chao, Youming Guo, Pengfei Li, Lee Chao

**Affiliations:** Department of Biochemistry and Molecular Biology, Medical University of South Carolina, Charleston, SC, USA

## Abstract

Oxidative stress has both detrimental and beneficial effects. Kallistatin, a key component of circulation, protects against vascular and organ injury. Serum kallistatin levels are reduced in patients and animal models with hypertension, diabetes, obesity, and cancer. Reduction of kallistatin levels is inversely associated with elevated thiobarbituric acid-reactive substance. Kallistatin therapy attenuates oxidative stress and increases endothelial nitric oxide synthase (eNOS) and NO levels in animal models. However, kallistatin administration increases reactive oxygen species formation in immune cells and bacterial killing activity in septic mice. High oxygen inhibits kallistatin expression via activating the JNK-FOXO1 pathway in endothelial cells. Conversely, mild oxygen/hyperoxia stimulates kallistatin, eNOS, and hypoxia-inducible factor-1 (HIF-1) expression in endothelial cells and in the kidney of normal mice. Likewise, kallistatin stimulates eNOS and HIF-1, and kallistatin antisense RNA abolishes oxygen-induced eNOS and HIF-1 expression, indicating a role of kallistatin in mediating mild oxygen's stimulation on antioxidant genes. Protein kinase C (PKC) activation mediates HIF-1-induced eNOS synthesis in response to hyperoxia/exercise; thus, mild oxygen through PKC activation stimulates kallistatin-mediated HIF-1 and eNOS synthesis. In summary, oxidative stress induces down- or upregulation of kallistatin expression, depending on oxygen concentration, and kallistatin plays a novel role in mediating oxygen/exercise-induced HIF-1-eNOS-NO pathway.

## 1. Introduction

Kallistatin was first identified in human plasma as a tissue kallikrein-binding protein (KBP) and characterized as a serine proteinase inhibitor (serpin) [1–3]. Tissue kallikrein (TK) is a serine proteinase that cleaves low molecular weight kininogen substrate to release vasodilating kinin peptides [4]. Kallistatin consists of two structural elements, an active site and a heparin-binding domain, which exert pleiotropic activities by regulating differential signaling pathways [5, 6]. Kallistatin through its active site forms a covalent complex with TK and inhibits TK activity [5, 7]. Kallistatin via its heparin-binding site interacts with cell surface heparan sulfate proteoglycans and thereby antagonizes the signaling pathways mediated by vascular endothelial growth factor, tumor necrosis factor-*α*, transforming growth factor-*β*, and Wnt [8–11]. Moreover, kallistatin exerts a wide spectrum of biological effects independent of TK. For example, kallistatin is a potent vasodilator unrelated to the tissue kallikrein-kinin system [12]. Transgenic mice overexpressing kallistatin have lower blood pressure compared to control mice and are resistant to lipopolysaccharide-induced mortality [13, 14]. Kallistatin is mainly expressed in the liver and is widely distributed in the kidney, heart, and blood vessel [15–18]. Circulating kallistatin levels are markedly reduced under pathological conditions, such as in hypertension, liver disease, sepsis, cardiac and renal injury, severe pneumonia, obesity, and cancer in patients and in animal models [19]. Kallistatin administration by gene or protein delivery alleviates hypertension, multiorgan damage, and cancer development by reducing oxidative stress, inflammation, angiogenesis, apoptosis, fibrosis, tumor growth, and metastasis in rodents [8, 20–25]. These findings indicate that kallistatin therapy has beneficial effects in various disease states.

Kallistatin belongs to the serpin family, which includes *α*1-antitrypsin and *α*1-antichymotrypsin [2]. In contrast to *α*1-antitrypsin, kallistatin is a negative acute-phase protein [26]. Kallistatin levels are markedly reduced in animals after endotoxin shock or experimental inflammation [26]. Oxidative stress downregulates kallistatin expression by activating c-Jun NH_2_-terminal kinase- (JNK-) dependent FOXO1 signaling in cultured endothelial cells [27]. However, hyperoxia treatment markedly stimulates kallistatin expression in breast cancer cells [28]. Moreover, kallistatin exhibits antioxidative actions. Kallistatin via its heparin-binding site antagonizes cytokine-induced reactive oxygen species (ROS) formation, and its active site is responsible for the upregulation of antioxidant gene expression in endothelial cells [9, 10, 29]. On the other hand, kallistatin stimulates ROS formation in immune cells, leading to marked bacterial killing activity in septic mice [28, 30]. Moreover, kallistatin's vasodilating activity is partly mediated by H_2_O_2_ formation [28]. Therefore, oxidative stress plays opposite roles in the regulation of kallistatin synthesis, and kallistatin possesses a dual role in modulating oxidative stress.

## 2. Reduced Circulating Kallistatin Levels Are Inversely Associated with Oxidative Stress

**Figure 1:**
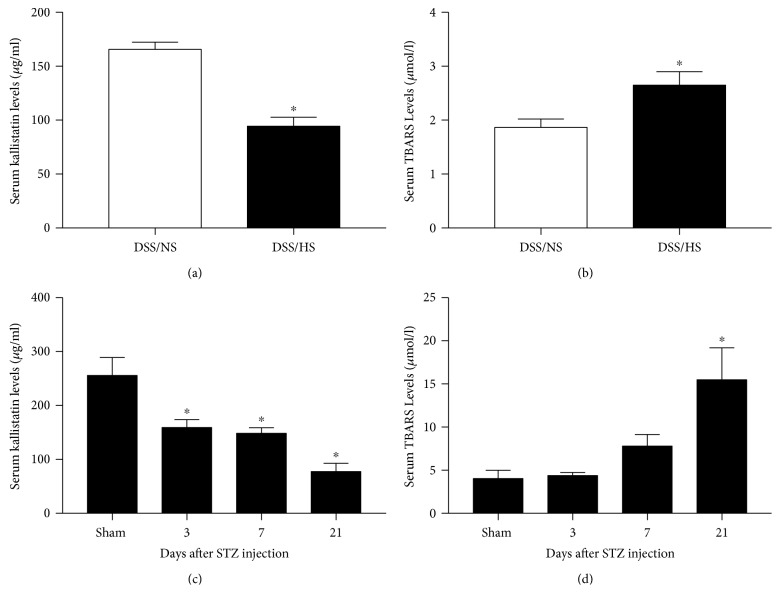
Circulating kallistatin levels are reduced, and TBARS levels are increased in DSS hypertensive rats and STZ-induced diabetic rats. ^∗^*P* < 0.05 versus control group.

Circulatory kallistatin levels are markedly reduced in spontaneous hypertensive and arterial hypertensive rats [15, 31, 32]. Reduced kallistatin levels are associated with increased oxidative organ damage in animal models of hypertension and cardiovascular and renal dysfunction. Likewise, plasma kallistatin levels are reduced in patients with liver disease, sepsis, pulmonary pneumonia, obesity, and cancer [19]. Moreover, serum kallistatin levels are decreased in Dahl salt-sensitive (DSS) hypertensive rats receiving a high-salt diet (HS), compared with DSS rats with a normal-salt diet (NS), and are reduced time dependently in rats receiving streptozotocin (STZ) injection, a model of diabetes (Figures 1(a) and 1(c)). Reduced kallistatin levels are inversely associated with elevated serum thiobarbituric acid reactive substances (TBARs, an indicator of lipid peroxidation) in hypertensive DSS rats and in STZ-induced diabetic rats (Figures 1(a), 1(b), 1(c), and 1(d)). These findings indicate that kallistatin levels are reduced in diseased states and, consequently, negatively associated with oxidative stress.

## 3. Kallistatin Treatment Reduces Oxidative Stress and Organ Damage

Oxidative stress is a key contributor to the pathogenesis of hypertension, inflammation, fibrosis, and multiorgan injury [33–35]. Kallistatin administration attenuates cardiovascular and renal damage associated with reduced superoxide formation, inflammation, and increased endothelial nitric oxide synthase (eNOS) and NO levels in animal models of acute and chronic myocardial damage and salt-induced hypertension [21, 36, 37]. Moreover, kallistatin treatment inhibits liver fibrosis via antioxidative stress [38]. Conversely, depletion of endogenous kallistatin by neutralizing antibody injection augments cardiovascular and renal injury, in conjunction with increased oxidative stress, inflammation, endothelial cell loss, and fibrosis in hypertensive rats [39]. Kallistatin acts as a potent antioxidant as it prevents oxidative NO inactivation induced by superoxide production in cultured renal epithelial tubular and mesangial cells, cardiomyocytes, myofibroblasts, endothelial cells, and endothelial progenitor cells (EPCs) [21, 27, 29, 37, 40]. Moreover, kallistatin exhibits antioxidant activity in cultured pterygium epithelial cells through inhibition of ROS formation [41]. Kallistatin's heparin-binding site is crucial for blocking tumor necrosis factor- (TNF-) *α*-induced NADPH oxidase activity and expression, and its active site is a key for stimulating the activity and expression of the antioxidant enzymes, eNOS, sirtuin 1 (SIRT1), and catalase in endothelial cells and EPCs [9, 10, 29]. Collectively, kallistatin protects against multiorgan damage through its antioxidative actions.

## 4. High Oxygen Downregulates Kallistatin Expression

**Figure 2:**
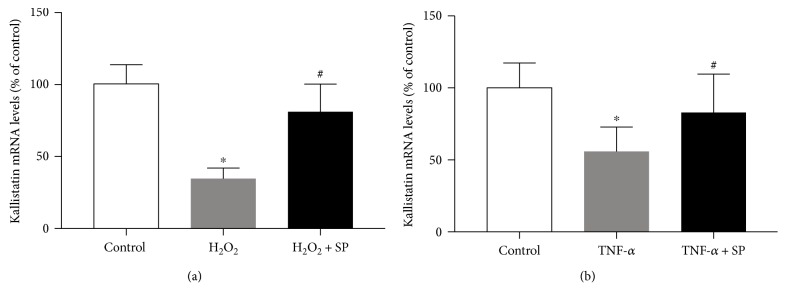
High H_2_O_2_ concentration (100 *μ*M, 24 hr) or TNF-*α* (10 ng/ml, 24 hr) inhibits kallistatin expression in human umbilical vein endothelial cells (HUVECs), which is blocked by SP, a JNK inhibitor. ^∗^*P* < 0.05 versus control group; ^#^*P* < 0.05 versus H_2_O_2_ or TNF-*α* group.

Oxidative stress effectively suppresses kallistatin expression *in vivo* and *in vitro*. Kallistatin synthesis is rapidly diminished in the liver of rats after endotoxin shock [26]. In cultured endothelial cells, high H_2_O_2_ concentration at 100 or 200 *μ*M significantly reduces kallistatin mRNA and protein levels by reverse transcription polymerase chain reaction and Western blot analysis [27]. Likewise, high H_2_O_2_ concentration (100 *μ*M, 24 hours) or TNF-*α* (10 ng/ml, 24 hours) markedly inhibits kallistatin expression, and the inhibitory effect of high H_2_O_2_ or TNF-*α* on kallistatin expression is abolished by SP, a JNK inhibitor in endothelial cells (Figures 2(a) and 2(b)). Moreover, knockout of FOXO1 with antisense RNA has been shown to block oxidative stress-mediated suppression of kallistatin synthesis in endothelial cells [27]. Therefore, oxidative stress activates the JNK-dependent FOXO1 signaling pathway leading to the inhibition of kallistatin expression.

## 5. Mild Oxygen or Hyperoxia Upregulates Kallistatin, HIF-1, and eNOS Expression

**Figure 3:**
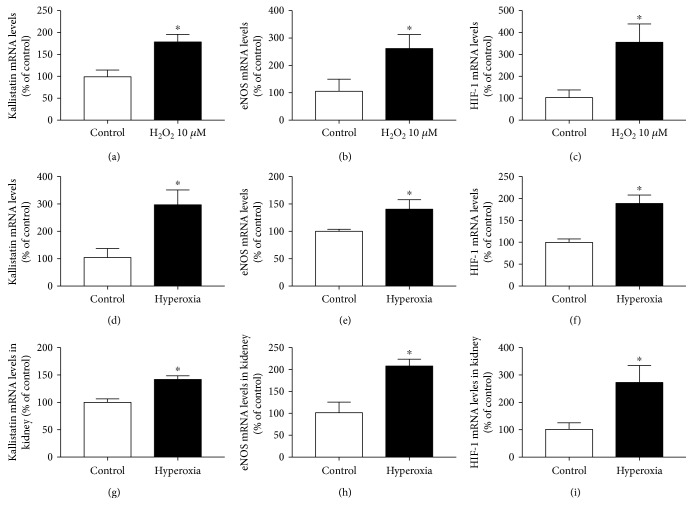
Mild H_2_O_2_ (10 *μ*M, 12 hr) or hyperoxia (95% O_2_/5% CO_2_, 6 hr for endothelial cells and 90 min for mice) stimulates kallistatin, eNOS, and HIF-1 synthesis in endothelial cells and in the kidney of mice. ^∗^*P* < 0.05 versus control group.

The physiology oxygen levels differ under *in vitro* and *in vivo* conditions. High H_2_O_2_ concentration (100 *μ*M) inhibits kallistatin expression (Figure 2). In contrast to high H_2_O_2_, low or mild H_2_O_2_ concentrations (1 to 30 *μ*M for 12 hours) dose dependently stimulate kallistatin synthesis in endothelial cells (data not shown). The stimulatory effect of H_2_O_2_ (10 *μ*M) on kallistatin expression is associated with increased eNOS and HIF-1 synthesis (Figures 3(a), 3(b), and 3(c)). Likewise, hyperoxia (95% O_2_/5% CO_2_ for 6 hours) stimulates the expression of kallistatin, eNOS, and HIF-1 in endothelial cells (Figures 3(d), 3(e), and 3(f)). In the *in vivo* hyperoxia model, mice were placed in a 95% O_2_/5% CO_2_ chamber for 90 minutes as described [42]. Hyperoxia treatment increases kallistatin, eNOS, and HIF-1 mRNA levels in the mouse kidney (Figures 3(g), 3(h), and 3(i)). Hyperbaric oxygen therapy has been shown to prolong survival of mice with systemic metastatic cancer, reduces the growth, and induces apoptosis in rat mammary tumors [43]. Indeed, our previous study showed that hyperoxia markedly induces kallistatin expression in MDA-MB-231 and MCF-7 breast cancer cells and kallistatin treatment inhibits tumor progression by inducing cancer cell apoptosis and autophagy [11, 28]. Therefore, mild oxygen or hyperbaric oxygen upregulates kallistatin, HIF-1, and eNOS expression and thus NO formation.

## 6. Kallistatin Mediates Mild Oxygen-Induced HIF-1 and eNOS Synthesis

**Figure 4:**
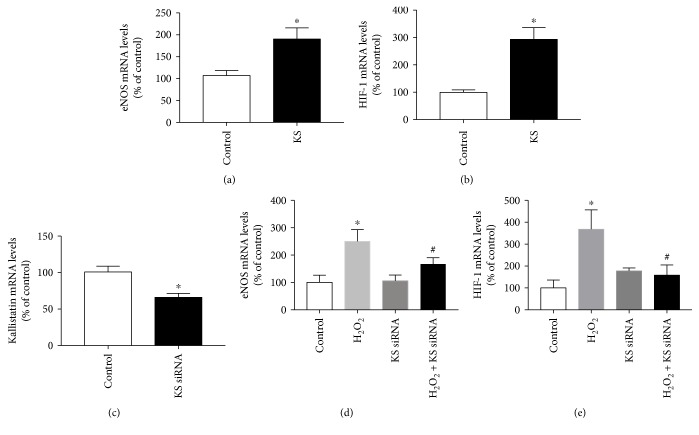
Kallistatin stimulates eNOS and HIF-1 expression and mediates mild oxygen-induced eNOS and HIF-1 synthesis in endothelial cells. ^∗^*P* < 0.05 versus control group; ^#^*P* < 0.05 versus H_2_O_2_ group.

In parallel with mild oxygen/hyperoxia, kallistatin is capable of stimulating eNOS and HIF-1 synthesis in endothelial cells (Figures 4(a) and 4(b)). Importantly, kallistatin not only elevates eNOS mRNA level but also increases eNOS activity and immunostaining, as well as NO formation in EPCs and endothelial cells [25, 40]. Moreover, kallistatin antisense RNA abolishes H_2_O_2_-induced eNOS and HIF-1 synthesis, indicating a role of kallistatin in mediating mild oxygen-induced eNOS and HIF-1 synthesis (Figures 4(c), 4(d), and 4(e)). Mild hyperoxia via protein kinase C (PKC) activation increases HIF-1 synthesis, and eccentric exercise induces HIF-1-mediated-eNOS expression [44–48]. Taken together, these findings indicate that kallistatin is a novel mediator of mild oxygen-induced HIF-1 and eNOS synthesis, and mild oxygen through PKC activation stimulates kallistatin-mediated HIF-1-eNOS pathway.

## 7. Kallistatin Protects against Vascular and Organ Injury by Mediating Oxygen/Exercise-Induced HIF-1-eNOS-NO Pathway

Physical activity and exercise training lower blood pressure in individuals with hypertension [49]. Moreover, exercise training exerts beneficial effects in diabetes and attenuates the decline of immune function associated with aging [50, 51]. Regular exercise prevents oxidative stress-related diseases, while acute exercise increases free-radical generation and oxidative injury in the elderly [52–54]. Older men who exercise on a regular basis do not demonstrate age-associated vascular oxidative stress [54]. Indeed, exercise prevents aging-induced decline of eNOS/NO in the aorta [55], and regular physical activity improves endothelial function in patients with coronary artery disease by increasing eNOS phosphorylation and in animals with elevated NO levels [56]. Likewise, regular aerobic exercise restores endothelial function in arteries of aged mice by reducing oxidative stress, increasing superoxide dismutase activity, and downregulating NADPH oxidase activity [57]. Kallistatin treatment attenuates vascular senescence and aging by suppression of oxidative stress and stimulation of antioxidant gene expression [29]. Moreover, kallistatin by stimulation of eNOS expression and activity and NO formation leads to blood pressure lowering, antioxidant and anti-inflammatory actions, and antiaging effect [9, 19, 21, 27, 29]. Therefore, regular exercise/mild oxygen increases kallistatin expression, leading to activation of HIF-1-eNOS-NO signaling. Collectively, kallistatin plays a protective role in vascular and organ injury by mediating oxygen/exercise-induced HIF-1-eNOS-NO pathway.

## 8. Conclusion

**Figure 5:**
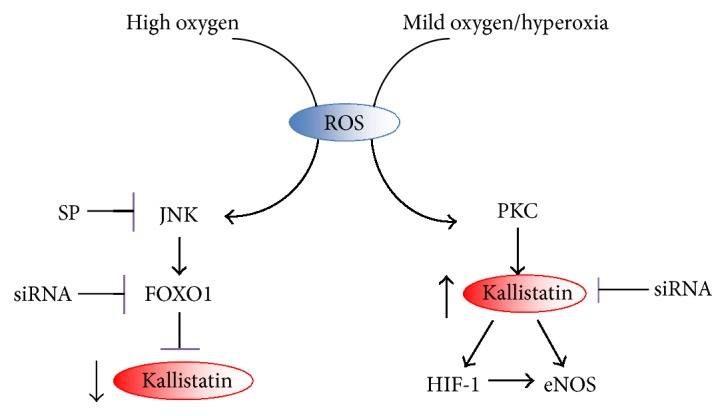
Signaling pathways mediated by high or mild oxygen on kallistatin regulation. High oxygen through ROS generation stimulates JNK/FOXO1 signaling activation, leading to kallistatin inhibition; mild oxygen/hyperoxia through PKC activation stimulates kallistatin-mediated HIF-1 and eNOS synthesis in endothelial cells.

Oxidative stress exerts opposite effects in regulating kallistatin expression. High H_2_O_2_ concentration (100 *μ*M) inhibits kallistatin expression via activating the JNK-dependent signaling pathway, while low or mild oxygen (H_2_O_2_ at 1 to 30 *μ*M) and mild hyperoxia via PKC activation stimulate kallistatin-mediated HIF-1 and eNOS synthesis (Figure 5). Moreover, kallistatin possesses both antioxidant and prooxidant actions. Reduced kallistatin levels in the circulation or tissues are inversely correlated with elevated superoxide levels, and kallistatin treatment attenuates oxidative stress and organ damage. In contrast, kallistatin stimulates ROS formation in immune cells and causes marked bacterial killing activity in septic mice. Kallistatin stimulates eNOS and HIF-1 synthesis, and depletion of kallistatin by kallistatin antisense RNA blocks mild oxygen-induced eNOS and HIF-1 synthesis. HIF-1 modulates eNOS expression induced by exercise training. Therefore, kallistatin acts as a novel mediator of oxygen/exercise-induced HIF-1-eNOS-NO pathway and protects against oxidative vascular and organ injury. In summary, these findings reveal two important messages: (1) oxygen at high or low concentration exerts opposing effects on kallistatin expression and (2) kallistatin, by its dual role in regulation of oxidative stress, displays beneficial effects in pathological conditions such as hypertension, cardiovascular and renal injury, sepsis, and cancer development.
